# Supplementary material for: Exploring the Use of Washington Group Questions to Identify People with Clinical Impairments Who Need Services including Assistive Products: Results from Five Population-Based Surveys
Source: Int J Environ Res Public Health. 2022 Apr 3;19(7):4304. doi: 10.3390/ijerph19074304 (PMC8998283; doi:10.3390/ijerph19074304)
Supplement: Supplementary file 1 [file ijerph-19-04304-s001.zip › ijerph-1624680-supplementary.pdf]

Table S1. Agreement between Washington Group question responses and impairment severity level in each functional domain

**Washington Group question responses**	**Impairment levels**				
	**None** N (%)	**Mild** N (%)	**Moderate** N (%)	**Severe** N (%)	**Blind (vision only) Profound (hearing only)** N (%)
			**Distance Vision Impairment**		
**India (n=3451)**					
No difficulty (n=2600)	2541 (97.73)	41 (1.58)	12 (0.46)	5 (0.19)	1 (0.04)
Some (n=763)	591 (77.46)	117 (15.33)	39 (5.11)	12 (1.57)	4 (0.52)
A lot (n=87)	37 (42.53)	5 (5.75)	20 (22.99)	16 (18.39)	9 (10.34)
Cannot do (n=1)	0 (0)	0 (0)	1 (100.00)	0 (0)	0 (0)
**Cameroon (n=3314)**					
No difficulty (n=2601)	2584 (99.35)		15 (0.58)	1 (0.04)	1 (0.04)
Some (n=659)	619 (93.93)		28 (4.25)	7 (1.06)	5 (0.76)
A lot (n=49)	29 (59.18)		12 (24.29)	2 (4.08)	6 (12.24)
Cannot do (n=5)	0 (0)		0 (0)	0(0)	5 (100.00)
**The Gambia (n=9180)***					
No difficulty (n=6650)	6218 (93.5)	134 (2.02)	236 (3.55)	45 (0.68)	17 (0.26)
Some (n=2351)	1586 (67.46)	178 (7.57)	443 (18.84)	108 (4.59)	36 (1.53)
A lot (n=160)	53 (33.13)	13 (8.13)	44 (27.5)	17 (10.63)	33 (20.63)
Cannot do (n=19)	0 (0)	0 (0)	1 (5.26)	0 (0)	18 (94.74)
			**Near Vision Impairment**		
**The Gambia (n=9175)*+**	***Can read***	***Cannot read***			
No difficulty (n=6646)	3756 (56.52)	2890 (43.48)			
Some (n=2350)	628 (26.72)	1722 (73.28)			
A lot (n=160)	21 (13.13)	139 (86.88)			
Cannot do (n=19)	0 (0)	19 (100)			
			**Hearing Impairment**		
**India (n=3253)****					

No difficulty (n=2829)	2703 (95.55)	100 (3.53)	22 (0.78)	4 (0.14)	0 (0)
Some (n=335)	228 (68.06)	57 (17.01)	41 (12.24)	7 (2.09)	2 (0.60)
A lot (n=86)	10 (11.63)	3 (3.49)	41 (47.67)	22 (25.58)	10 (11.63)
Cannot do (n=3)	0 (0)	0 (0)	0 (0)	0 (0)	3 (100)
**Cameroon (n=3005)****					
No difficulty (n=2582)	2430 (94.11)	117 (4.53)	34 (1.32)	1 (0.04)	0 (0)
Some (n=390)	295 (75.64)	48 (12.31)	36 (9.23)	8 (2.05)	3 (0.77)
A lot (n=33)	9 (27.27)	3 (9.09)	10 (30.30)	6 (18.18)	5 (15.15)
Cannot do (n=0)	0 (0)	0 (0)	0 (0)	0 (0)	0 (0)
**Chile (n=492)**					
No difficulty (n=283)	195 (68.90)	70 (24.73)	15 (5.30)	3 (1.06)	0 (0.00)
Some (n=173)	67 (38.73)	69 (39.88)	32 (18.50)	5 (2.89)	0 (0.00)
A lot (n=34)	5 (14.71)	4 (11.76)	18 (52.94)	7 (20.59)	0 (0.00)
Cannot do (n=2)	0 (0.00)	0 (0.00)	0 (0.00)	0 (0.00)	2 (100)
**Mobility: Musculoskeletal Impairment**					
**Cameroon (n=3308)**					
No difficulty (n=2466)	2332 (94.57)	91 (3.69)	40 (1.62)	3 (0.12)	
Some (n=740)	524 (70.81)	172 (23.24)	44 (5.95)	0 (0)	
A lot (n=98)	29 (29.59)	25 (25.51)	40 (40.82)	4 (4.08)	
Cannot do (n=4)	0 (0)	0 (0)	2 (50.00)	2 (50.00)	
**India (n=3439)**					
No difficulty (n=2707)	2460 (90.88)	227 (8.39)	16 (0.59)	4 (0.15)	
Some (n=611)	277 (45.34)	307 (50.25)	19 (3.11)	8 (1.31)	
A lot (n=107)	8 (7.48)	37 (34.58)	39 (36.45)	23 (21.50)	
Cannot do (n=14)	0 (0.00)	0 (0.00)	6 (42.86)	8 (5.14)	
**Turkey (n=3014)**					
No difficulty (n=2455)	2336 (88.18)	42 (38.18)	60 (32.43)	17 (24.29)	
Some (n=407)	281 (10.61)	33 (30.00)	72 (38.92)	21 (30.00)	
A lot (n=129)	30 (1.13)	34 (30.91)	45 (24.32)	20 (28.57)	
Cannot do (n=23)	2 (0.08)	1 (0.91)	8 (4.32)	12 (17.14)	

**Cognitive Impairment**			
**Chile (n=534)**			
No difficulty (n=96)	84 (87.50)	12 (12.50)	
Some (n=349)	313 (89.68)	36 (10.32)	
A lot (n=88)	67 (76.14)	21 (23.86)	
Cannot do (n=1)	0 (0)	1 (100.00)	

*8 participants were missing WG data; + Near vision test not possible for 5 participants; ** Limited to participants $\geq$ 4 years old with complete PTA; in Cameroon, 11 survey participants were missing WG data.

Table S2: Proportion of participants assessed to have a clinical impairment who need interventions as identified by Washington Group questions

	Washington Group Questions							
	Need surgical/medical intervention*				Need rehabilitation services/APs*			
**Domain**	**Total**	**No difficulty**	**Some+ Difficulty**	**A lot+ difficulty**	**Total**	**No difficulty**	**Some+ Difficulty**	**A lot+ difficulty**
	N	%	%	%	N	%	%	%
**Mild+ Visual Impairment <6/12**								
- India (n=282)	129	18%	82%	33%	153	26%	74%	5%
- The Gambia (n=1323)	785	27%	73%	13%	655	38%	62%	8%
**Moderate+ Visual Impairment <6/18**								
- Cameroon (n=82^)	63	16%	84%	32%	55	27%	73%	33%
- India (n=119)	103	15%	85%	40%	17	24%	76%	29%
- The Gambia (n=998)	726	26%	74%	14%	381	35%	65%	10%
**Near Vision Impairment**								
- The Gambia (n=4770)					4770	61%	39%	3%
**Mild+ Hearing Impairment**								
- Chile (n=225**)**	30	23%	77%	23%	211	40%	60%	13%
**Moderate+ Hearing Impairment**								
- Cameroon (n=103)	34	38%	62%	12%	55	33%	67%	25%
- Chile (n=82)	16	6%	94%	44%	78	23%	77%	31%
- India (n=153)	25	4%	96%	64%	140	18%	82%	50%
**Mobility: Mild+ MSI**								
- Cameroon (n=423)	276	30%	70%	17%	296	25%	75%	20%
- India (n=694)	349	36%	64%	18%	304	35%	65%	19%
- Turkey (n=365)	187	30%	70%	34%	318	31%	69%	34%
**Mobility: Moderate+ MSI**								
- Cameroon (n=135)	94	32%	68%	36%	96	22%	78%	44%
- India (n=123)	70	11%	89%	60%	66	14%	86%	62%
- Turkey (n=255)	136	28%	72%	34%	230	30%	70%	34%

* Some participants were assessed to need both surgical/medical and rehabilitation/APs interventions/services; ^2 participants who were clinically assessed to have moderate vision impairment did not have a cause of vision impairment assigned.

Table S3: Overall proportion of survey participants who were assessed to have a clinical impairment and need interventions

Domain	Washington Group Questions*		
	**Total population**	**Need medical/surgical intervention**	**Need rehabilitation services/AP**
	N	N (%)	N (%)
**Mild+ Visual Impairment <6/12**			
- India (n=282)	3451	129 (4%)	153 (4%)
- The Gambia (n=1323)	9180	785 (9%)	655 (7%)
**Moderate+ Visual Impairment <6/18**			
- Cameroon (n=82^)	3314	63 (2%)	55 (2%)
- India (n=119)	3451	103 (3%)	17 (<1%)
- The Gambia (n=998)	9180	726 (8%)	381 (4%)
**Mild+ Hearing Impairment**			
- Chile (n=225)	492	30 (6%)	211 (43%)
**Moderate+ Hearing Impairment**			
- Cameroon (n=103)	3005	34 (1%)	55 (2%)
- Chile (n=82)	492	16 (3%)	78 (16%)
- India (n=153)	3253	25 (1%)	140 (4%)
**Mobility: Mild+ MSI**			
- Cameroon (n=423)	3308	276 (8%)	296 (9%)
- India (n=694)	3439	349 (10%)	304 (9%)
- Turkey (n=365)	3014	187 (6%)	318 (11%)
**Mobility: Moderate+ MSI**			
- Cameroon (n=135)	3308	94 (3%)	96 (3%)
- India (n=123)	3439	70 (2%)	66 (2%)
- Turkey (n=255)	3014	136 (5%)	230 (8%)

*Analysis was restricted to those participants who answered Washington Group questions; ^2 participants who were clinically assessed to have moderate vision impairment did not have a cause of vision impairment assigned.

Table S4: Relationship between clinical impairment assessed need* for four priority assistive products (glasses, hearing aids, wheelchairs and protheses) and Washington Group responses in three functional domains.

	Washington Group Questions														
	VISION						HEARING			MOBILITY					
	Needs distance glasses			Needs near glasses			Needs hearing aid**			Needs wheelchair			Needs UL/LL prosthesis		
	No diff. ^<br>N (%)	Some+<br>N (%)	A lot+<br>N (%)	No diff.<br>N (%)	Some+<br>N (%)	A lot+<br>N (%)	No diff.<br>N (%)	Some+<br>N (%)	A lot+<br>N (%)	No diff.<br>N (%)	Some+<br>N (%)	A lot+<br>N (%)	No diff.<br>N (%)	Some+<br>N (%)	A lot+<br>N (%)
**Cameroon**															
Mild+										0<br>0%	4<br>100%	4<br>100%	0<br>0%	1<br>100%	0<br>0%
Moderate+	7<br>41%	10<br>59%	5<br>29%				10<br>28%	26<br>72%	8<br>22%	0<br>0%	4<br>100%	4<br>100%	0<br>0%	1<br>100%	0<br>0%
**Chile**															
Mild+							85<br>40%	126<br>60%	28<br>13%						
Moderate+							18<br>23%	60<br>77%	24<br>31%						
**India**															
Mild+	34<br>24%	110<br>76%	7<br>5%							1<br>50%	1<br>50%	1<br>50%	1<br>50%	1<br>50%	1<br>50%
Moderate+	3<br>19%	13<br>81%	5<br>31%				20<br>19%	85<br>81%	54<br>51%	1<br>50%	1<br>50%	1<br>50%	1<br>50%	1<br>50%	1<br>50%
**The Gambia**															
Mild+	214<br>40%	315<br>60%	22<br>4%	2643<br>66%	1359<br>34%	59<br>1%									
Moderate+	163<br>39%	260<br>61%	17<br>4%												
**Turkey**															
Mild+										0	9	6	0	2	2

		0%	100%	67%	0%	100%	100%
Moderate+		0	9	6	0	2	2
		0%	100%	67%	0%	100%	100%

Abbreviations: diff.= difficulty, UL/LL=upper limb/lower limb; * ”Need” includes both ”unmet need” and ”undermet need” for each assistive product; ^ Denominator includes participants who needed specific assistive product and who completed Washington Group questions in the respective functional domain; ** Hearing aid need includes all participants who need a referral for audiological services and likely hearing aid need.
